# Isolation and Genetic Characterization of *Toxoplasma gondii* from a Patas Monkey (*Erythrocebus patas*) in China

**DOI:** 10.3390/genes14081606

**Published:** 2023-08-10

**Authors:** Liulu Yang, Hongjie Ren, Niuping Zhu, Shilin Xin, Gaohui Mao, Yiheng Ma, Junbao Li, Qunchao Liang, Yurong Yang

**Affiliations:** 1 Veterinary Pathology, Henan Agricultural University, Zhengzhou 450000, China; 2 Zhengzhou Zoo, Zhengzhou 450000, China; 3 Henan Yinji Jiabao Amusement Park Management Co., Ltd., Xinmi 452300, China

**Keywords:** toxoplasmosis, non-human primates, patas monkey (*Erythrocebus patas*), isolation, ToxoDB genotype #9

## Abstract

Many cases of *Toxoplasma gondii* infection have been reported worldwide in non-human primates (NHPs), especially in captive New World monkeys. However, few studies on toxoplasmosis in Old World monkeys have been conducted. In this study, serological and molecular biological analyses were carried out to look for *T. gondii* antibodies and *T. gondii* infection in 13 NHPs from China. *T. gondii* infection was confirmed in 8 NHP cases. *T. gondii* antibodies were detected in 1/5 New World monkeys and in 4/7 Old World monkeys. *T. gondii* DNA was detected in 3/5 New World monkeys and 5/7 Old World monkeys. The one ring-tailed lemur was negative for both antibodies and DNA of *T. gondii*. The most common clinical manifestations of *T. gondii* infection were malaise, poor appetite, emaciation, and foamy nasal discharge. The most common histopathological findings were interstitial pneumonia, necrotic hepatitis, necrotizing myocarditis, lymphadenitis, and necrotic splenitis. One viable *T. gondii* strain was successfully isolated from the myocardium of a patas monkey (*Erythrocebus patas*) by bioassay in mice. *T. gondii* tachyzoites were obtained from cell cultures and were designated as TgMonkeyCHn2. The genotype of this strain belongs to ToxoDB genotype #9, and the allele of ROP18/ROP5 gene was 3/6. TgMonkeyCHn2 tachyzoites were avirulent in Swiss mice. To our knowledge, this is the first report of fatal toxoplasmosis in a patas monkey. *T. gondii* infection in patas monkeys may indicate environmental contamination by oocysts. The patas monkey is a new host record for *T. gondii*.

## 1. Introduction

*Toxoplasma gondii* is a zoonotic intracellular apicomplexan protozoan parasite that infects most warm-blooded animals, including non-human primates (NHPs) and humans [1,2]. *T. gondii* infection in captive animals is of interest because many species, especially NHPs and some Australian marsupials, often die due to severe toxoplasmosis [3,4,5,6,7]. New World monkeys, such as squirrel monkeys, saki monkeys, howler monkeys, and ring-tailed lemurs, are more susceptible to *T. gondii* than Old World monkeys and often develop fatal disease [6,8,9,10]. To date, several *T. gondii* genotypes from viable strains (n = 7) in NHPs have been identified worldwide, namely ToxDB #163, ToxDB #4, ToxDB #206, and four mixed genotypes [11,12,13,14]. The objective of the present investigation was to estimate the *T. gondii* infection status of captive NHPs from China by testing IgG antibodies, isolation, and genetic characterization of *T. gondii*. Additionally, possible sources of *T. gondii* infection in the zoos were evaluated to prevent future occurrences of toxoplasmosis.

## 2. Materials and Methods

### 2.1. Sample Collection and Clinical Case Report

Between 2020 and 2021, 13 NHPs died in zoos in Henan province, China (Table 1). Whole or partial tissue samples, including brain, heart, liver, spleen, lung, kidney, tongue, leg muscles, diaphragm, and intestines, were collected from 13 monkeys and transported to the veterinary pathology laboratory, Henan Agricultural University (Zhengzhou, Henan, China) for pathological diagnosis. Details of these cases are provided in Table 1.

### 2.2. T. gondii Antibody and T. gondii DNA Were Detected in Monkey Tissues

The modified agglutination test (MAT) was used to detect the anti-*T. gondii* antibody in the heart fluid of the monkeys [15]. Formalin-treated tachyzoites of *T. gondii* were obtained from the University of Tennessee Research Foundation (Knoxville, TN, USA; https://utrf.tennessee.edu/, accessed on 1 February 2020). *T. gondii*-positive mouse sera were provided by Dr. J. P. Dubey (Beltsville, MD, USA) and were used as the reference sera. The heart juice samples were tested at a ratio of 1:2, and the dilution was then doubled to a titer of 1: 256. Positive samples were further diluted from 1:200 to the final titer. Negative and positive controls were included in each plate. DNA was extracted from monkey tissues and pepsin-digested tissues using a DNA extraction kit (DP304; Tiangen Biotec Co., Tiangen, China). *T. gondii* DNA was checked using the specific primer pair TOX5/TOX8 (5′-CGCTGCAGACACAGTGCATCTGGATT-3′ and 5′-CCCA GCTGCGTCTGTCGGGAT-3′) in PCR assays [16]. PCR reaction process: initial denaturation at 94 °C for 2 min; 35 cycles of amplification (94 °C for 1 min, 60 °C for 1 min, and 72 °C for 1 min) and final extension at 72 °C for 10 min, 6 °C forever. The PCR product length was estimated to be 450 bp, and both negative and positive controls were included.

### 2.3. Histopathological Analysis

Tissue samples from all monkeys were fixed in 10% (*v*/*v*) neutral-buffered formalin. The tissues were processed using conventional histological techniques and embedded in paraffin. Paraffin sections (5 μm in thickness) of the samples were prepared and stained with hematoxylin and eosin (HE). Immunohistochemical (IHC) staining was performed on monkeys suspected to be infected with *T. gondii* [17,18]. The rabbit anti-*T. gondii* polyclonal antibody was provided by Dr. Dubey (Beltsville, MD, USA Agricultural Research Service, USDA). The rabbit-specific HRP/DAB immunohistochemical assay kit was purchased from Abcam (ab64264). Brain tissue sections from mice infected with *T. gondii* (VEG strain) were used as positive controls for IHC staining (provided by Dr. Dubey, ARS, USDA).

### 2.4. Isolation of Viable T. gondii from Monkey Tissues Using Mice Bioassay

Sample tissues from nine NHPs were bioassayed in mice as per previously described methods [1,17] (Table 1). Briefly, tissue samples (50 g, including 15 g brain, 15 g heart, 10 g leg muscle, 5 g diaphragm, and 5 g tongue) from monkeys were digested in a pepsin solution and inoculated into Swiss mice (n = 2–5) or gamma interferon (IFN-γ^−/−^) knockout mice (n = 1–2). The survival time of IFN-γ^−/−^ mice infected with *T. gondii* is short, which is an ideal animal model of acute toxoplasmosis [19,20,21,22]. In addition, the homogenized tissues of case#16 (red howler monkey: myocardium, diaphragm, and skeletal muscles), case#26 (white-cheeked gibbon: pleural and peritoneal effusion) were inoculated directly into Swiss mice. Tissue samples (brain, heart, spleen, lung, liver, kidney, skeletal muscle, lymph nodes) from case#18 (squirrel monkey), case#24 (mona monkey), case#25 (mona monkey), and case#28 (ring-tailed lemur) were homogenized and inoculated into gamma interferon (IFN-γ^−/−^) knockout mice (n = 1) or Swiss mice (n = 3–5), respectively. Clinical signs were recorded daily. At 30 days after inoculation (dpi), the serum anti-*T. gondii* antibodies were detected by MAT at 1:25 and 1:200 dilutions. Tachyzoites or cysts were examined in the lungs or brains of dead and euthanized mice. If no cysts or tachyzoites were found in mouse tissues, then mouse lung, brain, heart, and tongue homogenates were subpassaged into a new group of mice.

### 2.5. In Vitro Cultivation and Genotyping

Tissues (brain, lung, or mesenteric lymph nodes) from *T. gondii*-positive mice were seeded in Vero cell culture flasks (RPMI 1640, 3% fetal bovine serum, 37 °C, and 5% CO_2_) [1]. DNA was extracted from the cell culture-derived tachyzoites. *T. gondii* strain genotyping was performed using 10 PCR-RFLP genetic markers (SAG1, SAG2, SAG3, BTUB, GRA6, c22-8, c29-2, L358, PK1, and Apico) as previously described by Su et al. [23]. Genotyping of the virulence genes ROP18 and ROP5 was performed as previously described [24,25,26]. All batches contained *T. gondii* reference DNA.

### 2.6. Evaluation of the Virulence of the T. gondii Strain Isolated from Monkeys

We evaluated the virulence of *T. gondii* isolated from monkeys in Swiss mice [27]. *T. gondii* tachyzoites were collected from cell cultures. They were counted in a disposable hemocytometer and diluted 10-fold from 10^−1^ to 10^−4^ to reach an endpoint of <1 tachyzoite. Then, <1, 10^0^, 10^1^, 10^2^, and 10^3^ tachyzoites were intraperitoneally inoculated into four Swiss mice at each dilution. Clinical signs were recorded, and the mice were monitored daily. After 30 days, all surviving mice were bled and tested for anti-*T. gondii* IgG antibodies by MAT with titers between 1:25 and 1:200. Mice were euthanized at 60 dpi, after which their brains were examined and tissue cysts enumerated. All tissues were fixed in 10% (*v*/*v*) neutral-buffered formalin. Virulence was evaluated according to the percentage of dead *T. gondii*-positive mice.

### 2.7. Statistical Analysis

Statistical analysis was performed using GraphPad Prism 8.0 software (GraphPad Software Inc., San Diego, CA, USA). Data were analyzed using the Chi-squared test or Fisher’s exact test. Statistical significance was set at *p* < 0.05.

## 3. Results

### 3.1. Clinical Signs, Pathological Evaluation, and Immunohistochemistry

**Figure 1:**
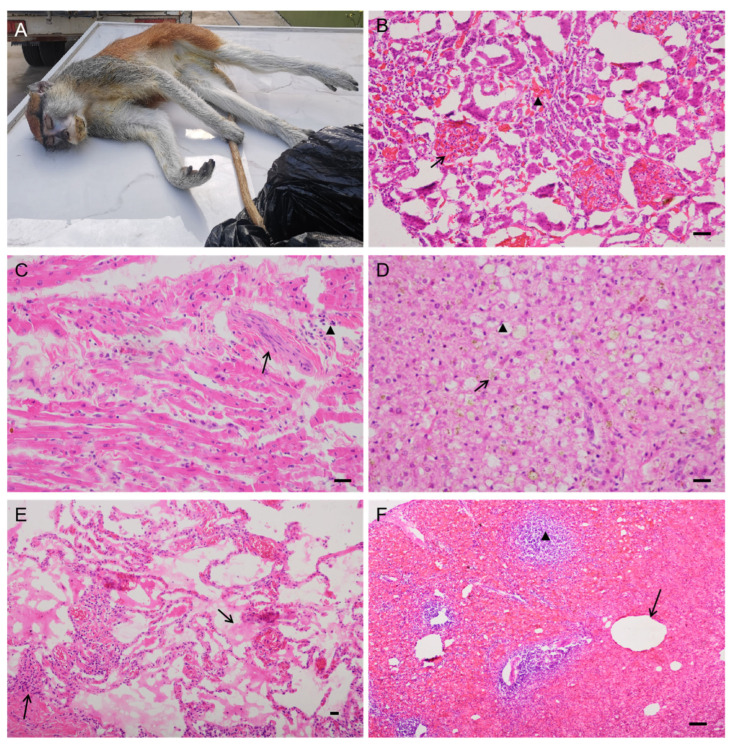
Patas monkey (Case#21) and its microscopic appearance of major organs. (**A**) Patas monkey died on 25 December 2020; (**B**) moderate interstitial hemorrhage (arrowhead) with glomerular hemorrhage (arrow) and severe glomerulonephritis, kidney, HE; (**C**) focal myocardial fibrosis (arrow) and multifocal inflammatory cell infiltration (arrowhead) with myocardial necrosis, heart, HE; (**D**) multifocal inflammatory cell infiltration with hepatocyte necrosis, mild and diffuse hemosiderosis (arrow), mild steatosis (arrowhead), liver, HE; (**E**) moderate edema with fibrin (hyaline membrane) (arrow), mild hemorrhage; mild lymphocyte infiltration and interstitial pneumonia, lung, HE; (**F**) severe hemorrhage with gas gangrene (arrow); acute necrotizing splenitis and lymphoid depletion (arrowhead); spleen, HE; bar = 50 μm.

Samples were collected from 13 NHPs between 2020 and 2021 (Table 1). *T. gondii* parasites were not found in IHC- and HE-stained tissue sections of these monkeys. The most common clinical manifestations were emaciation (46%, 6/13), malaise (31%, 4/13), and poor appetite (31%, 4/13). Moreover, renal insufficiency (69%, 9/13), age-related atrophy (54%, 7/13), necrotic myocarditis (39%, 5/13), interstitial pneumonia (31%, 2/13), necrotic enteritis (31%, 2/13), necrotic metritis (8%, 1/13), suppurative cystitis (8%, 1/13), lymphadenitis (8%, 1/13), and necrotic splenitis (8%, 1/13) were observed. Some histopathological lesions are shown in Figure 1.

### 3.2. T. gondii Examination by MAT and PCR

Heart fluid and tissue samples from 13 NHPs were tested for *T. gondii* serology and molecular biology; all monkeys were adults. PCR confirmed that eight monkeys were infected with *T. gondii*.

*T. gondii* antibodies were detected in 38% (5/13) of the monkeys, with a titer of 1:4 in one case, 1:8 in one case, 1:64 in two cases, and 1:3200 in one case. The MAT showed that 57% (4/7) of Old World monkeys had *T. gondii* antibodies, while 20% (1/5) of New World monkeys had *T. gondii* antibodies. However, the difference in *T. gondii* antibodies between Old World and New World monkeys was not significant (*p* = 0.2929). The proportion of female monkeys with *T. gondii* antibodies was more (57%, 4/7) than the proportion of male monkeys with *T. gondii* antibodies (17%, 1/6), although this difference was not significant (*p* = 0.2657).

*T. gondii* DNA was detected in 8 of the 13 NHPs. The proportion of Old World monkeys infected with the *T. gondii* was more (71%, n = 5/7) than that of New World monkeys (60%, n = 3/5), and one ring-tailed lemur was negative for *T. gondii* DNA. Parasite DNA was mainly observed in the spleen, tongue, lymph nodes, lungs, and kidneys but less frequently in the heart, skeletal muscles, intestines, and other organs (Table 1). In addition, only four PCR-positive cases showed *T. gondii* antibody transformation (≥1:4) (Table 1).

### 3.3. Viable T. gondii Was Isolated from Monkey Tissue Samples Using Mouse Bioassays and Genetic Characterization

**Figure 2:**
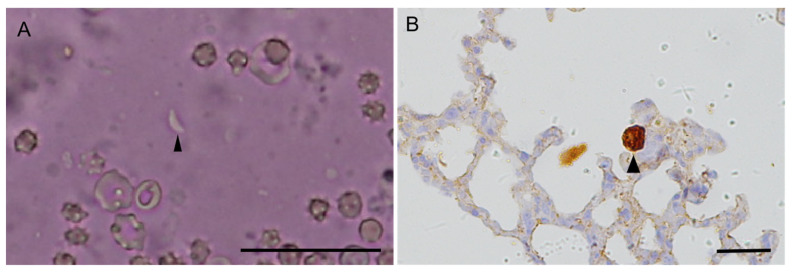
(**A**) *T. gondii* tachyzoites (arrowhead) were found in the lungs of IFN-γ^−/−^ mouse, 18 dpi, smear, unstained; (**B**) aggregation of tachyzoites with a parasitophorous vacuole (arrowhead) was detected in the lungs of IFN-γ^−/−^ mouse, 24 dpi, IHC; bar = 50 μm.

Tissues from 9 out of 13 NHPs were individually tested via bioassay in mice (Table 1). In the Tox#20-31 group, four mice were inoculated with digestive fluid from the myocardial and skeletal muscles of case#21 (patas monkey); two IFN-γ^−/−^ mice (M#511, M#586) died after showing signs of toxoplasmosis at 18–19 dpi and *T. gondii* tachyzoites were observed in the lungs (Figure 2). The remaining two Swiss mice had seroconverted antibodies for *T. gondii* at 30 dpi, and cysts (30 cysts from M#512 and 110 cysts from M#458) were observed in the brain at 317 dpi. The *T. gondii* strain from the lung of M#511 was successfully propagated in cell cultures (17 DPI) and was designated as TgMonkeyCHn2. *T. gondii* tachyzoites were found and confirmed in the mouse lungs by IHC staining (Figure 2). Genotyping the isolate of TgMonkeyCHn2 indicated that it was ToxoDB#9 using 10 PCR-RFLP markers (Table 2). The ROP18/ROP5 allele of TgMonkeyCHn2 isolates was 3/6.

In the other eight cases, none of the mice (n = 2–5) had antibodies against *T. gondii*, and no parasite was observed in mice tissues at 30–393 dpi.

### 3.4. Virulence Evaluation of TgMonkeyCHn2 by Mice

As shown in Table 3, 10^3^ TgMonkeyCHn2 tachyzoites infected all Swiss mice, as confirmed by MAT at 30 dpi. Most mice were asymptomatic within 60 dpi after intraperitoneal inoculation with tachyzoites. However, two mice inoculated with 10^3^ TgMonkeyCHn2 tachyzoites died of toxoplasmosis at 46 and 54 dpi, respectively, and *T. gondii* tachyzoites were detected in lung tissue. *T. gondii* cysts (0–60 cysts) were detected in mice brains when euthanized at 67 dpi.

## 4. Discussion

This study investigated *T. gondii* infection in 13 captive monkeys who died of suspected toxoplasmosis or other diseases in China zoos. Eight monkeys were confirmed to be infected with *T. gondii* by PCR. Four of the eight monkeys in which *T. gondii* DNA was detected were serologically negative (titer < 1:4). This may be because of acute infection and infected monkeys may die of the disease before they can produce detectable IgG titers. Most monkey toxoplasmosis cases result in widespread histopathological lesions and intralesional *T. gondii* organisms [28,29,30,31,32]. Unfortunately, neither immunohistochemical staining nor HE staining of 1 cm^2^ tissue sections from 13 monkeys revealed *T. gondii* cysts or tachyzoites. Moreira et al. also confirmed a case of toxoplasmosis in a black-and-gold howler monkey by increasing anti-*T. gondii* antibody titers (IFAT, 1:16–1:256 for 36 days); blood was tested for *T. gondii* nucleic acid, and *T. gondii* was isolated from the liver and heart, but no cysts or tachyzoites in the lung and liver were detected by HE and IHC staining [33]. Here, PCR (8/13) was more sensitive than histopathology (0/13) and serology (5/13), especially for acute *T. gondii* infection or parasitemia. We also conclude that serology is still an important method for diagnosing *T. gondii* infection in NHPs in addition to molecular methods; blood cell samples should be taken to allow for nucleic acid detection.

MAT has been widely used to detect anti-*T. gondii* IgG antibodies in serum or body fluids of animals and its effectiveness has been demonstrated with *T. gondii* isolated from pigs, lambs, chickens, and monkeys [34,35,36,37]. However, the validity of MAT in NHPs remains unclear. Previous studies using MAT at 1:16 and 1:20 dilutions have detected toxoplasmosis or isolated viable *T. gondii* strains in monkeys [31,32,37]. Here, a viable *T. gondii* strain was isolated from the striated muscles of case#21 (patas monkey), with a *T. gondii* antibody titer of 1:3200, and *T. gondii* infection was also confirmed by PCR detection in tissues (spleen, lung, kidney, and mesenteric lymph nodes). Previous studies have also detected high *T. gondii* antibody titers (1:1024, 1:10,240, 1:3200, and ≥1:500) in four other patas monkeys by MAT [11,38,39,40,41]. This may be related to their ground-dwelling habits, as they have more exposure to *T. gondii* oocysts than monkeys with tree-dwelling habits.

In this study, from the eight infected *T. gondii* NHPs, the parasite DNA was mainly observed in the spleen, lungs, tongue, lymph nodes, kidney, heart, skeletal muscles, and pancreas. Viable TgMonkeyCHn2 isolates were successfully obtained from the myocardial and skeletal muscles of a patas monkey at a titer of 1:3200 via a mouse bioassay. However, *T. gondii* DNA was not detected in the myocardial or skeletal muscles in this case. This result suggests that the density of *T. gondii* parasites in the striated muscle of this patas monkey was low. In a previous report, *T. gondii* DNA was detected in the lungs, liver, heart, and brain of squirrel monkeys that died of toxoplasmosis [31]. In addition, *T. gondii* DNA has been isolated from the liver and brain of a captive ring-tailed lemur with toxoplasmosis [32]. These results indicate the relatively low density of *T. gondii* parasites in the organs of some monkeys diagnosed with toxoplasmosis.

Although the higher susceptibility of New World monkeys to *T. gondii* has not been fully elucidated, their specific immune response to the parasite may be due to the lack of feline contact for more than 20 million years during the evolution of New World monkeys. *T. gondii* can infect Old World monkeys, and they can survive, and there have been no reports of clinical natural toxoplasmosis in Old World NHPs [11].

According to the summary of *T. gondii* viable strains isolated from humans and animals in China (Table S1), at least more than 16 ToxoDB RFLP-genotypes were found. These include ToxoDB RFLP-genotype #9, #1–#6, #10, #17–#18, #20, #204–#205, #292, #319, and mix genotypes. Among these viable *T. gondii* strains isolated, 65% were ToxoDB #9 (*Chinese 1*); 7% were ToxoDB #2; and 4% were ToxoDB #3, #10, and #205. *T. gondii* ToxoDB #9 was in clade D and haplogroup 13; beyond China, it is also distributed in Mexico [42,43], Colombia, Vietnam, Sri Lanka, West Indies, and Brazil [44,45,46,47,48,49]. Most of ToxoDB #9 strains were avirulent [50]; however, they have also been confirmed to be associated with clinical toxoplasmosis in humans [51,52], pigs [48], and mice [53]. The natural host range of ToxoDB #9 involves cats, humans, pigs, sheep, tigers, and cheetahs [50,51,53,54,55,56] (Table S1), and patas monkeys were also demonstrated to be natural intermediate hosts of *T. gondii* ToxoDB #9 in the present study.

Dubey et al. reported a high success rate of genotyping of *T. gondii* (ToxoDB#3, ToxoDB#9, ToxoDB#11, ToxoDB#21, ToxoDB#36) from tissues of NHPs that died of acute toxoplasmosis [11], indicating that we could genotype the samples directly. However, none of these samples were successfully genotyped in this study because of their low DNA concentration.

TgMonkeyCHn2 was non-lethal, and the number of brain cysts was low in Swiss mice. Recent studies have shown that the genetic diversity and population structure of *T. gondii* can affect its virulence [57]. In this study, TgMonkeyCHn2 may be an avirulent phenotype, which was predicted by ROP18 (allele 3) and ROP5 (allele 6) [24,25]. The virulence phenotype was supported by mouse infection (low cyst formation rate and avirulence). The ROP18/ROP5 type of TgMonkeyCHn1 (3/6) has also been confirmed to be non-lethal in Swiss mice [37].

Most primates in zoos were not serologically tested for *T. gondii* at the time of introduction. Thus, it is impossible to determine the time and source of infection. Patas monkeys are omnivorous, and their diet consists of fruits, seeds, leaves, flowers, buds, bird eggs, insects, and arthropods. The source of *T. gondii* infection in the patas monkey is unclear. Vertical transmission of *T. gondii* could occur in ring-tailed lemurs via endogenous transplacental transmission [58]; however, this is unclear in other monkey species. The patas monkey may have acquired *T. gondii* by ingesting oocysts from the feces of felids or mechanical transporters (insects, arthropods, zookeepers, or cleaning tools). This indicated that *T. gondii* oocysts contaminate the habitat environment (water and soil) of monkeys. The high prevalence of antibodies against *T. gondii* in stray cats (50%), captive tigers (80%), servals (100%), caracals (67%), and cheetahs (100%) in central China supports this hypothesis [59,60,61].

The present study is the first to isolate *T. gondii* from a patas monkey and the first molecularly confirmed case of *T. gondii* infection in patas monkeys, providing direct evidence that patas monkeys are intermediate hosts of *T. gondii*. Patas monkeys could be used as good sentinel animals for monitoring environmental *T. gondii* contamination.

## Figures and Tables

**Table 1:** Background and isolation of *Toxoplasma gondii* from non-human primates in China.

Case No.	Received Date	Species	Sex, Age	Pathology No.	Clinical Signs	Pathological Findings	*T. gondii*		Mice Bioassay	
							MAT ^a^	PCR ^b^	Swiss	IFN-γ^−/−^
New World Monkeys										
Case#16	27 February 2020	Red howler monkey (*Alouatta seniculus*)	F, adult	3022	Asymptomatic	Age-related atrophy, fatty degeneration of the liver, lipofuscin deposition in multiple organs.	1:64	H, Sk, P, I ^c^	0/5 ^e^	nd
Case#17	15 April 2020	Red howler monkey (*Alouatta seniculus*)	F, adult	3024	Weight loss, lost two teeth	Renal insufficiency, necrotic splenitis, hypoproteinemia.	<1:2	Sp	nd	nd
Case#18	9 Novemebr 2020	Squirrel monkey (*Saimiri sciureus*)	M, adult	3080	Malaise, poor appetite, emaciation. Foamy nasal and oral discharge, died 2 days after onset of symptoms	Necrotic glomerulonephritis, necrotic enteritis, pulmonary congestion, interstitial pneumonia.	<1:2	- ^**d**^	0/4	0/1
Case#19	13 March 2021	Red howler monkey (*Alouatta seniculus*)	F, adult	3130	Malaise, poor appetite, emaciation	Suppurative pneumonia, glomerulonephritis, hypoproteinemia.	<1:2	Sp, Lu, T, Ly	nd	nd
Case#20	31 July 2021	White-faced saki monkey (*Pithecia pithecia*)	M, adult	3203	Malaise, poor appetite, emaciation	Cardiac insufficiency, hydropericardium, hydrothorax, ascites, glomerulonephritis, skeletal muscle atrophy.	<1:2	-	nd	nd
Old World Monkeys										
Case#21	25 December 2020	Patas monkey (*Erythrocebus patas*)	M, adult	3108	Tail gangrene	Multiple organ gas gangrene, renal insufficiency, cardiac insufficiency, lipofuscin deposition in the liver, interstitial pneumonia.	1:3200	Sp, Lu, K, Ly	2/2	2/2
Case#22	9 January 2020	Hamadryas baboon (*Papio hamadryas*)	F, adult	3112	One week after the delivery of the fetus	Necrotizing metritis, necrotizing myocarditis.	1:64	-	nd	0/2
Case#23	17 February 2021	Hamadryas baboon (*Papio hamadryas*)	M, 24 years	3121	Leader, asymptomatic	Parenchymatous myocarditis, renal insufficiency, fatty degeneration of the liver.	<1:2	-	0/3	nd
Case#24	15 April 2021	Mona monkey (*Cercopithecus mona*)	F, adult	3148	Emaciation	Renal insufficiency, skeletal muscle atrophy.	1:8	Sp	0/5	nd
Case#25	15 April 2021	Mona monkey (*Cercopithecus mona*)	F, adult	3149	Emaciation	Renal insufficiency, skeletal muscle atrophy, necrotizing hepatitis.	1:4	K, T, Ly	0/3	nd
Case#26	4 August 2021	White-cheeked Gibbon (*Nomascus leucogenys*)	M, adult	3205	Malaise, poor appetite, depression, fever, four limbs, neck and abdomen edema; died 35 days after treatment	Cachexia, age-related atrophy, necrotic enteritis, renal insufficiency, and acute hemorrhagic lymphadenitis.	<1:2	H, Sp, K, T, Ly, Sk, D	0/2	nd
Case#27	10 August 2021	Black-and-white colobus (*Colobus polykomos*)	M, adult	3208	Emaciation	Necrotic myocarditis, multiple organ atrophy.	<1:2	T, P	nd	nd
Lemuriformes										
Case#28	22 September 2021	Ring-tailed lemur (*Lemur catta*)	F, adult	3218	White fluid comes out of the vulva	Suppurative hemorrhagic cystitis, urethral calculus.	<1:2	-	0/3	nd

nd: Experiment not done. ^a^: Modified agglutination test, titer 1:2–1:12,800; ^b^: Polymerase chain reaction; ^c^: *T. gondii* nucleic acid was detected in tissue; H: Heart; Sp: Spleen; Lu: Lung; K: Kidney; T: Tongue; Ly: Mesenteric lymph nodes; Sk: Skeletal muscle; D: Diaphragm; P: Pancreas; I: Intestines; ^d^: Negative result; ^e^: No. of mice infected/No. of mice inoculated.

**Table 2:** Genotypes of *Toxoplasma gondii* isolates from patas monkey in China according to PCR-RFLP of 10 markers and virulence proteins.

Isolated ID	SAG1	(3′ + 5′) SAG2	Alt SAG2	SAG3	BTUB	GRA6	C22-8	C29-2	L358	PK1	Apico	ROP18	ROP5	ToxoDB Genotype
GT1, reference	I	I	I	I	I	I	I	I	I	I	I	1	1	#10
PTG, reference	II/III	II	II	II	II	II	II	II	II	II	II	2	2	#1
CTG, reference	II/III	III	III	III	III	III	III	III	III	III	III	3	3	#2
TgCgCa1, reference	I	II	II	III	II	II	II	u-1	I	u-2	I	2	5	#66
MAS, reference	u-1	I	II	III	III	III	u-1	I	I	III	I	4	4	#17
TgCatBr5, reference	I	III	III	III	III	III	I	I	I	u-1	I	4	4	#19
TgCatBr64, reference	I	I	u-1	III	III	III	u-1	I	III	III	I	3	3	#111
TgRsCr1, reference	u-1	I	II	III	I	III	u-2	I	I	III	I	3	3	#52
TgMonkeyCHn2	u-1	II	II	III	III	II	II	III	II	II	II	3	6	#9, this study

**Table 3:** Evaluation of the virulence of *Toxoplasma gondii* TgMonkeyCHn2 strain in Swiss mice.

No. of Tachyzoites	No. of Mice Infection/ No. of Mice Inoculation (%)	Days of Survival/Number of Mice	No. of Brain Cysts
10^3^	4/4 (100%)	≥60 dpi/2, 46 dpi/1, 54 dpi/1	2.5 ± 2.5
10^2^	2/4 (50%)	≥60 dpi/4	Not found
10^1^	2/4 (50%)	≥60 dpi/4	35.0 ± 25.0
1	2/4 (50%)	≥60 dpi/4	Not found
<1	0/4 (-)	≥60 dpi/4	Not found
Blank control	0	≥60 dpi/4	Not found

## Data Availability

The data supporting the findings of this study are available from the corresponding author upon request. The TgMonkeyCHn2 isolates were cryopreserved and available for further analysis.

## Supplementary Materials

The following supporting information can be downloaded at: https://www.mdpi.com/article/10.3390/genes14081606/s1, Table S1: Summary of viable *T. gondii* isolates (n = 169) from animals and humans in China [62,63,64,65,66,67,68,69,70,71,72,73,74,75,76,77].

## References

[1] Dubey J.P. (2010). Toxoplasmosis of Animals and Humans.

[2] Dubey J.P. (2022). Toxoplasmosis of Animals and Humans.

[3] Basso W., Venturini M.C., Moré G., Quiroga A., Bacigalupe D., Unzaga J.M., Larsen A., Laplace R., Venturini L. (2007). Toxoplasmosis in captive Bennett’s wallabies (*Macropus rufogriseus*) in Argentina. Vet. Parasitol..

[4] Bermúdez R., Faílde L.D., Losada A.P., Nieto J.M., Quiroga M.I. (2009). Toxoplasmosis in Bennett’s wallabies (*Macropus rufogriseus*) in Spain. Vet. Parasitol..

[5] Bouer A., Werther K., Catão-Dias J.L., Nunes A.L. (1999). Outbreak of toxoplasmosis in *Lagothrix lagotricha*. Folia Primatol..

[6] Dubey J.P., Kramer L.W., Weisbrode S.E. (1985). Acute death associated with *Toxoplasma gondii* in ring-tailed lemurs. J. Am. Vet. Med. Assoc..

[7] Epiphanio S., Guimarães M.A., Fedullo D.L., Correa S.H., Catão-Dias J.L. (2000). Toxoplasmosis in golden-headed lion tamarins (*Leontopithecus chrysomelas*) and emperor marmosets (*Saguinus imperator*) in captivity. J. Zoo Wildl..

[8] Dietz H.H., Henriksen P., Bille-Hansen V., Henriksen S.A. (1997). Toxoplasmosis in a colony of New World monkeys. Vet. Parasitol..

[9] Carme B., Ajzenberg D., Demar M., Simon S., Dardé M.L., Maubert B., de Thoisy B. (2009). Outbreaks of toxoplasmosis in a captive breeding colony of squirrel monkeys. Vet. Parasitol..

[10] Oh H., Eo K.Y., Gumber S., Hong J.J., Kim C.Y., Lee H.H., Jung Y.M., Kim J., Whang G.W., Lee J.M. (2018). An outbreak of toxoplasmosis in squirrel monkeys (*Saimiri sciureus*) in South Korea. J. Med. Primatol..

[11] Dubey J.P., Murata F.H.A., Cerqueira-Cézar C.K., Kwok O.C.H., Yang Y., Su C. (2021). Recent epidemiologic, clinical, and genetic diversity of *Toxoplasma gondii* infections in non-human primates. Res. Vet. Sci..

[12] Huang M., Li G.F., Zhi G.L., Zhao C.Y., Liu K.X., Zuo K.J., Lan H., Chen X.J., Yuan Z.G. (2018). Isolation and identification of *Toxoplasma gondii* from black-crown squirrel monkey (*Saimiri vanzolinii*) of South China. Chin. Vet. Sci..

[13] Reis Amendoeira M.R., Arruda I.F., Moreira S.B., Ubiali D.G., da Silva Barbosa A., Jesus Pena H.F., Barbosa Pereira A.H., Nascimento da Silveira C., Bonifácio T.F., Clemes Y.S. (2022). Isolation and genetic characterization of *Toxoplasma gondii* from a captive black-and-gold howler monkey (*Alouatta caraya* Humboldt, 1812) in Brazil. Int. J. Parasitol. Parasites Wildl..

[14] Yang F., Li K.X., Xu C.Z., Shan F., Chen W., Peng S.M., Li W.P., Li G.Q. (2017). Multi-locus genotyping of the *Toxoplasma gondii* isolated from captive ring-tailed lemur (*Lemur catta*) in China. Chin. J. Wildl..

[15] Dubey J.P., Desmonts G. (1987). Serological responses of equids fed *Toxoplasma gondii* oocysts. Equine Vet. J..

[16] Schares G., Herrmann D.C., Beckert A., Schares S., Hosseininejad M., Pantchev N., Vrhovec M.G., Conraths F.J. (2008). Characterization of a repetitive DNA fragment in *Hammondia hammondi* and its utility for the specific differentiation of *H. hammondi* from *Toxoplasma gondii* by PCR. Mol. Cell. Probes.

[17] Su R., Dong H., Li T., Jiang Y., Yuan Z., Su C., Zhang L., Yang Y. (2019). *Toxoplasma gondii* in four captive kangaroos (*Macropus spp.*) in China: Isolation of a strain of a new genotype from an eastern grey kangaroo (*Macropus giganteus*). Int. J. Parasitol. Parasites Wildl..

[18] Hsu S.M., Raine L., Fanger H. (1981). Use of avidin–biotin–peroxidase complex (ABC) in immunoperoxidase techniques: A comparison between ABC and unlabelled antibody (PAP) procedures. J. Histochem. Cytochemi..

[19] Dubey J.P., Ferreira L.R., Martins J., McLeod R. (2012). Oral oocyst-induced mouse model of toxoplasmosis: Effect of infection with *Toxoplasma gondii* strains of different genotypes, dose, and mouse strains (transgenic, out-bred, in-bred) on pathogenesis and mortality. Parasitology.

[20] Jones T.C., Bienz K.A., Erb P. (1986). In vitro cultivation of *Toxoplasma gondii* cysts in astrocytes in the presence of gamma interferon. Infect. Immun..

[21] Sa Q., Ochiai E., Tiwari A., Perkins S., Mullins J., Gehman M., Huckle W., Eyestone W.H., Saunders T.L., Shelton B.J. (2015). Cutting edge: IFN-γ produced by brain-resident cells is crucial to control cerebral infection with *Toxoplasma gondii*. J. Immunol..

[22] Yano A., Mun H.S., Chin M., Norose K., Hata K., Kobayashi M., Aosai F., Iwakura Y. (2002). Roles of IFN-gamma on stage conversion of an obligate intracellular protozoan parasite, *Toxoplasma gondii*. Int. Rev. Immunol..

[23] Su C., Shwab E.K., Zhou P., Zhu X.Q., Dubey J.P. (2010). Moving towards an integrated approach to molecular detection and identification of *Toxoplasma gondii*. Parasitology.

[24] Shwab E.K., Jiang T., Pena H.F., Gennari S.M., Dubey J.P., Su C. (2016). The ROP18 and ROP5 gene allele types are highly predictive of virulence in mice across globally distributed strains of *Toxoplasma gondii*. Int. J. Parasitol..

[25] Shwab E.K., Jiang T., Pena H.F., Gennari S.M., Dubey J.P., Su C. (2016). Corrigendum to "The ROP18 and ROP5 gene allele types are highly predictive of virulence in mice across globally distributed strains of *Toxoplasma gondii*" (*Int. J. Parasitol.*
**2016**, *46*, 141–146). Int. J. Parasitol..

[26] Rêgo W., Costa J., Baraviera R., Pinto L.V., Bessa G.L., Lopes R., Vitor R. (2017). Association of ROP18 and ROP5 was efficient as a marker of virulence in atypical isolates of *Toxoplasma gondii* obtained from pigs and goats in Piauí, Brazil. Vet. Parasitol..

[27] Saraf P., Shwab E.K., Dubey J.P., Su C. (2017). On the determination of *Toxoplasma gondii* virulence in mice. Exp. Parasitol..

[28] Cedillo-Peláez C., Rico-Torres C.P., Salas-Garrido C.G., Correa D. (2011). Acute toxoplasmosis in squirrel monkeys (*Saimiri sciureus*) in Mexico. Vet. Parasitol..

[29] Dubey J.P., Hodgin E.C., Hamir A.N. (2006). Acute fatal toxoplasmosis in squirrels (*Sciurus carolensis*) with bradyzoites in visceral tissues. J. Parasitol..

[30] Paula N.F., Dutra K.S., Oliveira A.R., Santos D.O.D., Rocha C.E.V., Vitor R.W.A., Tinoco H.P., Costa M.E.L.T.D., Paixão T.A.D., Santos R.L. (2020). Host range and susceptibility to *Toxoplasma gondii* infection in captive neotropical and Old-world primates. J. Med. Primatol..

[31] Salant H., Weingram T., Spira D.T., Eizenberg T. (2009). An outbreak of toxoplasmosis amongst squirrel monkeys in an Israeli monkey colony. Vet. Parasitol..

[32] Spencer J.A., Joiner K.S., Hilton C.D., Dubey J.P., Toivio-Kinnucan M., Minc J.K., Blagburn B.L. (2004). Disseminated toxoplasmosis in a captive ring-tailed lemur (*Lemur catta*). J. Parasitol..

[33] Moreira S.B., Pereira A.H.B., Pissinatti T.A., Arruda I.F., de Azevedo R.R.M., Schiffler F.B., Amendoeira M.R.R., Dos Santos A.F.A., Pissinatti A., Workgroup Outbreak (2022). Subacute multisystemic toxoplasmosis in a captive black-and-gold howler monkey (*Alouatta caraya*) indicates therapy challenging. J. Med. Primatol..

[34] Dubey J.P., Thulliez P., Weigel R.M., Andrews C.D., Lind P., Powell E.C. (1995). Sensitivity and specificity of various serologic tests for detection of *Toxoplasma gondii* infection in naturally infected sows. Am. J. Vet. Res..

[35] Dubey J.P., Sundar N., Hill D., Velmurugan G.V., Bandini L.A., Kwok O.C.H., Majumdar D., Su C. (2008). High prevalence and abundant atypical genotypes of *Toxoplasma gondii* isolated from lambs destined for human consumption in the USA. Int. J. Parasitol..

[36] Dubey J.P., Laurin E., Kwowk O.C. (2016). Validation of the modified agglutination test for the detection of *Toxoplasma gondii* in free range chickens by using cat and mouse bioassay. Parasitology.

[37] Xin S., Jiang N., Yang L., Zhu N., Huang W., Li J., Zhang L., Su C., Yang Y. (2022). Isolation, genotyping and virulence determination of a *Toxoplasma gondii* strain from non-human primate from China. Transboundary Emerg. Dis..

[38] Alvarado-Esquivel C., Gayosso-Dominguez E.A., Villena I., Dubey J.P. (2013). Seroprevalence of *Toxoplasma gondii* infection in captive mammals in three zoos in Mexico City, Mexico. J. Zoo Wildl. Med..

[39] Cano-Terriza D., Almería S., Caballero-G´omez J., Díaz-Cao J.M., Jim´enez-Ruiz S., Dubey J.P., García-Bocanegra I. (2019). Serological survey of *Toxoplasma gondii* in captive nonhuman primates in zoos in Spain. Comp. Immunol. Microbiol. Infect. Dis..

[40] Chen R.F., Tang Y., Wang S.K., Chen X.L., Hu L.Y., Chen M.Z., Zhang J., Huang C.Q. (2015). Serological investigation of chlamydia and *Toxoplasma gondii* infection in wild animals from Fuzhou Zoo. Anim. Husb. Vet. Med..

[41] Marujo R.B., Langoni H., Ullmann L.S., Pellizzaro M., Neto R.N.D., Camossi L.G., Teixeira R.F., Nunes A.V., da Silva R.C., Menozzi B.D. (2017). *Toxoplasma gondii* antibodies and related risk factors in mammals at Sorocaba zoo, Sao Paulo, Brazil. Semin. Cienc. Agrar..

[42] Alvarado-Esquivel C., Rajendran C., Ferreira L.R., Kwok O.C., Choudhary S., Alvarado-Esquivel D., Rodríguez-Peña S., Villena I., Dubey J.P. (2011). Prevalence of *Toxoplasma gondii* infection in wild birds in Durango, Mexico. J. Parasitol..

[43] Dubey J.P., Velmurugan G.V., Alvarado-Esquivel C., Alvarado-Esquivel D., Rodríguez-Peña S., Martínez-García S., González-Herrera A., Ferreira L.R., Kwok O.C., Su C. (2009). Isolation of *Toxoplasma gondii* from animals in Durango, Mexico. J. Parasitol..

[44] Chaichan P., Mercier A., Galal L., Mahittikorn A., Ariey F., Morand S., Boumediene F., Udonsom R., Hamidovic A., Murat J.B. (2017). Geographical distribution of *Toxoplasma gondii* genotypes in Asia: A link with neighboring continents. Infect. Genet. Evol..

[45] Dubey J.P., Cortés-Vecino J.A., Vargas-Duarte J.J., Sundar N., Velmurugan G.V., Bandini L.M., Polo L.J., Zambrano L., Mora L.E., Kwok O.C. (2007). Prevalence of *Toxoplasma gondii* in dogs from Colombia, South America and genetic characterization of *T*. gondii isolates. Vet. Parasitol..

[46] Dubey J.P., Huong L.T., Sundar N., Su C. (2007). Genetic characterization of *Toxoplasma gondii* isolates in dogs from Vietnam suggests their South American origin. Vet. Parasitol..

[47] Dubey J.P., Rajapakse R.P.V.J., Wijesundera R.R.M.K.K., Sundar N., Velmurugan G.V., Kwok O.C.H., Su C. (2007). Prevalence of *Toxoplasma gondii* in dogs from Sri Lanka and genetic characterization of the parasite isolates. Vet. Parasitol..

[48] Olinda R.G., Pena H.F., Frade M.T., Ferreira J.S., Maia L.Â., Gennari S.M., Oliveira S., Dantas A.F., Riet-Correa F. (2016). Acute toxoplasmosis in pigs in Brazil caused by *Toxoplasma gondii* genotype *Chinese 1*. Parasitol. Res..

[49] Shwab E.K., Zhu X.Q., Majumdar D., Pena H.F.J., Gennari S.M., Dubey J.P., Su C. (2014). Geographical patterns of *Toxoplasma gondii* genetic diversity revealed by multilocus PCR-RFLP genotyping. Parasitology.

[50] Yang Y.R., Feng Y.J., Lu Y.Y., Dong H., Li T.Y., Jiang Y.B., Zhu X.Q., Zhang L.X. (2017). Antibody detection, isolation, genotyping, and virulence of *Toxoplasma gondii* in captive felids from China. Front. Microbiol..

[51] Zhou P., Zhang H., Lin R.Q., Zhang D.L., Song H.Q., Su C., Zhu X.Q. (2009). Genetic characterization of *Toxoplasma gondii* isolates from China. Parasitol. Int..

[52] Zhou P., Chen Z., Li H.L., Zheng H., He S., Lin R.Q., Zhu X.Q. (2011). *Toxoplasma gondii* infection in humans in China. Parasit. Vectors.

[53] Yang Y., Dong H., Su R., Jiang N., Li T., Su C., Yuan Z., Zhang L. (2019). Direct evidence of an extra-intestinal cycle of *Toxoplasma gondii* in tigers (*Panthera tigris*) by isolation of viable strains. Emerg. Microbes Infect..

[54] Dubey J.P., Zhu X.Q., Sundar N., Zhang H., Kwok O.C., Su C. (2007). Genetic and biologic characterization of *Toxoplasma gondii* isolates of cats from China. Vet. Parasitol..

[55] Wang D., Liu Y., Jiang T., Zhang G., Yuan G., He J., Su C., Yang N. (2016). Seroprevalence and genotypes of *Toxoplasma gondii* isolated from pigs intended for human consumption in Liaoning province, northeastern China. Parasit. Vectors.

[56] Yang Y., Feng Y., Yao Q., Wang Y., Lu Y., Liang H., Zhu X., Zhang L. (2017). Seroprevalence, isolation, genotyping, and pathogenicity of *Toxoplasma gondii* strains from sheep in China. Front. Microbiol..

[57] Shwab E.K., Saraf P., Zhu X.Q., Zhou D.H., McFerrin B.M., Ajzenberg D., Schares G., Hammond-Aryee K., van Helden P., Higgins S.A. (2018). Human impact on the diversity and virulence of the ubiquitous zoonotic parasite *Toxoplasma gondii*. Proc. Natl. Acad. Sci. USA.

[58] Juan-Sallés C., Mainez M., Marco A., Sanchís A.M. (2011). Localized toxoplasmosis in a ring-tailed lemur (*Lemur catta*) causing placentitis, stillbirths, and disseminated fetal infection. J. Vet. Diagn. Investig..

[59] Yang Y., Ying Y., Verma S.K., Cassinelli A.B., Kwok O.C., Liang H., Pradhan A.K., Zhu X.Q., Su C., Dubey J.P. (2015). Isolation and genetic characterization of viable *Toxoplasma gondii* from tissues and feces of cats from the central region of China. Vet. Parasitol..

[60] Dong H., Su R., Li T., Su C., Zhang L., Yang Y. (2019). Isolation, genotyping and pathogenicity of a *Toxoplasma gondii* strain isolated from a Serval (*Leptailurus serval*) in China. Transbound Emerg. Dis..

[61] Jiang N., Xin S., Li J., Su C., Zhang L., Yang Y. (2020). Isolation and characterization of *Toxoplasma gondii* from captive caracals (*Caracal caracal*). Int. J. Parasitol. Parasites Wildl..

[62] Jiang Y., Xin S., Ma Y., Zhang H., Yang X., Yang Y. (2023). Low prevalence of *Toxoplasma gondii* in sheep and isolation of a viable strain from edible mutton from central China. Pathogens.

[63] Chen Z.W., Gao J.M., Huo X.X., Wang L., Yu L., Halm-Lai F., Xu Y.H., Song W.J., Hide G., Shen J.L. (2011). Genotyping of *Toxoplasma gondii* isolates from cats in different geographic regions of China. Vet. Parasitol..

[64] Hou Z., Zhou Y., Liu D., Su S., Zhao Z., Xu J., Tao J. (2018). Genotyping and virulence analysis of *Toxoplasma gondii* isolates from a dead human fetus and dead pigs in Jiangsu province, Eastern China. Acta Parasitol..

[65] Jiang N., Su R., Jian F., Su C., Zhang L., Jiang Y., Yang Y. (2020). *Toxoplasma gondii* in lambs of China: Heart juice serology, isolation and genotyping. Int. J. Food Microbiol..

[66] Li Y.N., Nie X., Peng Q.Y., Mu X.Q., Zhang M., Tian M.Y., Min S.J. (2015). Seroprevalence and genotype of *Toxoplasma gondii* in pigs, dogs and cats from Guizhou province, Southwest China. Parasit. Vectors.

[67] Liang Y., Chen J., Meng Y., Zou F., Hu J., Esch G.W. (2016). Occurrence and genetic characterization of GRA6 and SAG2 from *Toxoplasma gondii* oocysts in cat feces, Kunming, China. Southeast Asian J. Trop. Med. Public Health.

[68] Qian W., Wang H., Su C., Shan D., Cui X., Yang N., Lv C., Liu Q. (2012). Isolation and characterization of *Toxoplasma gondii* strains from stray cats revealed a single genotype in Beijing, China. Vet. Parasitol..

[69] Ren H., Yang L., Zhu N., Li J., Su C., Jiang Y., Yang Y. (2022). Additional evidence of tigers (*Panthera tigris altaica*) as intermediate hosts for *Toxoplasma gondii* through the isolation of viable strains. Int. J. Parasitol. Parasites Wildl..

[70] Wang L., Chen H., Liu D., Huo X., Gao J., Song X., Xu X., Huang K., Liu W., Wang Y. (2013). Genotypes and mouse virulence of *Toxoplasma gondii* isolates from animals and humans in China. PLoS ONE.

[71] Wang L., Cheng H.W., Huang K.Q., Xu Y.H., Li Y.N., Du J., Yu L., Luo Q.L., Wei W., Jiang L. (2013). *Toxoplasma gondii* prevalence in food animals and rodents in different regions of China: Isolation, genotyping and mouse pathogenicity. Parasit. Vectors.

[72] Yang L., Xin S., Zhu N., Li J., Su C., Yang Y. (2023). Two viable *Toxoplasma gondii* isolates from red-necked wallaby (*Macropus rufogriseus*) and red kangaroo (*M. rufus*). Parasitol. Int..

[73] Yang L., Ren H., Zhu N., Mao G., Li J., Su C., Jiang Y., Yang Y. (2023). Epidemiology and isolation of viable *Toxoplasma gondii* strain from macropods. Heliyon.

[74] Yang Y., Dong H., Su R., Li T., Jiang N., Su C., Zhang L. (2019). Evidence of red panda as an intermediate host of *Toxoplasma gondii* and *Sarcocystis* species. Int. J. Parasitol. Parasites Wildl..

[75] Yang Y., Jiang N., Xin S., Zhang L. (2020). *Toxoplasma gondii* infection in white spoonbills (*Platalea leucorodia*) from Henan Province, China. Emerg. Microbes Infect..

[76] Zhou Y., Zhang H., Cao J., Gong H., Zhou J. (2013). Isolation and genotyping of *Toxoplasma gondii* from domestic rabbits in China to reveal the prevalence of type III strains. Vet. Parasitol..

[77] Zhang W., Liu J.S., Ma Y.W., Lu S. (1987). *Toxoplasma gondii* isolated from a dead deformed fetus. Chin. J. Zoonoses.

