# Supplementary material for: Isolation and Genetic Characterization of Toxoplasma gondii from a Patas Monkey (Erythrocebus patas) in China
Source: Genes (Basel). 2023 Aug 10;14(8):1606. doi: 10.3390/genes14081606 (PMC10454590; doi:10.3390/genes14081606)
Supplement: Supplementary file 1 [file genes-14-01606-s001.zip › genes-2506408-supplementary.pdf]

Table S1 Summary of viable *T. gondii* isolates (n=169) from animals and human in China

Province	species	No. of animls	Seroprevalence	Bioassay of tissues[b]			Genotype ToxoDB#	References
			Test, cut-off titer (%)	Number	Positive	Strain ID		
Beijing	Stray cat	64	MAT, 20 (58)	23	11	TgCatBj1-11	All #9	Qian et al. (2012)
Anhui (22), Hubei (23), Guangdong (20), Shanxi (24)	cat	211	ND	89	14	TgCtys1,2 TgCtwh1-8, TgCtgd1,2 TgCtsx1,2	12 #9, 2 #205	Chen et al. (2011)
ND	Human cat	ND	ND	ND	23	TgHuZSE, TgHuZS2, TgHuAh1-2, TgCtwh9-12,14,19 TgCtxz1-8, TgCtsd1-5	15 #9, 2 #10, 1 #1, 1 #204, 4 #205	Wang et al. (2013a)
Guizhou (23+17), Anhui (24), Hubei (13)	Cat,Pig, Chicken,vole	77	ND	B	11	TgCtgy1-5, TgCtgy1-4 TgCksz1, TgVowh1	10 #9, 1 #205	Wang et al. (2013b)
Guangdong	Market cat	34	MAT, 40(79)	27	17	TgCtPRC1-17	2#18, 15#9	Dubey et al. (2007)
Guangdong	Human, Pig, Cat, Sheep	ND	ND	ND	17	SH, CN,NT, TgC1-TgC8 GYS,PYS,ZC,ZS,ZS1,QHO	3 #10, 1 #3, 12 #9, 1 # MIX	Zhou et al. (2009)
Shanghai	rabbit	77	MAT,25(23)	18	1	SHR	SAG2, SAG3, GRA6, BTUB All type III	Zhou et al. (2013)
Liaoning	pig	2063	MAT,25(11.3)	233	23	TgPigCn3-2, 3-12, 5-5,5-6,5-7, 8-16, 8-18,10-34,14-56,19-24, 22-10,27-8, 29-5, 33-6,40-10, 58-14, 61-11,69-2, 69-14,77-2,80-11,80-25,80-27	1#3, 1#3 or #1, 13#9	Wang et al., 2016
Kunming	feline feces	115	Morphology, (4)	ND	1	KM isolate	type II	Liang et al., 2016
Guizhou	Pig, cat	70+19	ELISA, (49+12)	ND	5	TGGZ1-3(pig), TGGZ4-5(cat)	All #9	Li et at., 2015
Jiangsu	human fetus pigs	ND	ND	ND	3	KS, YZ-1, YZ-2	1 #10, 2#9	Hou et al., 2018 Zhang et al., 1987
Henan[c], Zhejiang	Stray cat, pet cat	42	MAT, 100(50)	42	8+1=9	TgCatCHn1-3, TgCatCZg1-5 TgCatCHn4 (feces)	6 #9, 1 #1, 1 #2, 1 #17	Yang et al., 2015
Henan	Hospital cat	28	MAT,25(7)	8,	1	TgCatCHn5	#9, 1	Yang et al., 2017a
Henan	Sheep	840	MAT,25(20.7)	26	2	TgSheepHn1-2	All #9	Yang et al., 2017b

Henan	Sheep	166	MAT,100(25)	67	11	TgSheepHn3-13	7 #2, 4 #4	Jiang et al., 2020a
Henan	Sheep	224	MAT,100(1.8)	4	1	TgSheepHn14	#3	Jiang et al., 2023
Henan	Kangaroo	4	MAT,25(75)	4	1	TgRooCHn1	#292	Su et al., 2019
Henan	Kangaroo	3	MAT,25(67)	3	2	TgRooCHn2-3	#3, #2	Yang et al., 2023a
Henan	Kangaroo	26	MAT,25(50)	6	1	TgRooCHn4	#3	Yang et al., 2023b
Henan	Red panda	14	MAT,25(14)	8	1	TgRedpandaCHn1	#20	Yang et al., 2019a
Henan	Serval	1	MAT,25(100)	1	1	TgServalCHn1	#20	Dong et al., 2019
Henan	Tiger	10	MAT,25(80)	6	2	TgTigerCHn1-2	All #9	Yang et al., 2019b
Henan	Tiger	4	MAT,25(100)	4	2	TgTigerCHn3-4	#20, #2	Ren et al., 2022
Henan	Caracal	2	MAT,25(100)	2	1	TgCaracalCHn1	#2	Jiang et al., 2020b
Henan	Caracal	1	MAT,25(100)	1	1	TgCaracalCHn2	#5	Ren HJ et al., unpublished
Henan	white spoonbills	7	MAT,4(29)	2	1	TgSpoonbillCHn1	#2	Yang et al., 2020
Henan	Monkey	15	MAT,8(47)	9	1	TgMonkeyCHn1	Mix	Xin et al., 2022
Henan	Monkey	13	MAT,8(62)	9	1	TgMonkeyCHn2	#9	This study
Henan	Monkey	9	MAT,8(22)	3	1	TgMonkeyCHn3	#6	Ma YH et al., unpublished
Henan	Cheetah	6	MAT,25(100)	6	2	TgCheetahCHn1-2	#319, #9	Zhu NP et al., unpublished
Henan	Fur seal	4	MAT,25(50)	4	1	TgfursealCHn1	#5	Mao GH et al., unpublished
Total	110 #9, 12 #2, 7 #205, 6 #10, 6 #3, 4 #4, 3 #20, 2 #1, 2 #18, 2 #5, 1 #6, 1 #204, 1 #17, 1 #292, 1 #319							

ND: experiment not done;

MAT: modified agglutination test;

ELISA: enzyme-linked immunosorbent assay.

References

1. Chen ZW, Gao JM, Huo XX, Wang L, Yu L, Halm-Lai F, Xu YH, Song WJ, Hide G, Shen JL, Lun ZR. Genotyping of *Toxoplasma gondii* isolates from cats in different geographic regions of China. Vet Parasitol. 2011, 183(1-2): 166-70.
2. Dong H, Su R, Li T, Su C, Zhang L, Yang Y. Isolation, genotyping and pathogenicity of a Toxoplasma gondii strain isolated from a Serval (Leptailurus serval) in China. Transbound Emerg Dis. 2019, 66(4): 1796-1802.
3. Dubey JP, Zhu XQ, Sundar N, Zhang H, Kwok OC, Su C. Genetic and biologic characterization of *Toxoplasma gondii* isolates of cats from China. Vet Parasitol. 2007, 145(3-4): 352-356.
4. Hou Z, Zhou Y, Liu D, Su S, Zhao Z, Xu J, Tao J. Genotyping and virulence analysis of *Toxoplasma gondii* isolates from a dead human fetus and dead pigs in Jiangsu province, Eastern China. Acta Parasitol. 2018, 63(2): 397-411.
5. Jiang N, Su R, Jian F, Su C, Zhang L, Jiang Y, Yang Y. *Toxoplasma gondii* in lambs of China: heart juice serology, isolation and genotyping. Int J Food Microbiol. 2020a, 322: 108563.
6. Jiang N, Xin S, Li J, Su C, Zhang L, Yang Y. Isolation and characterization of *Toxoplasma gondii* from captive caracals (*Caracal caracal*). Int J Parasitol Parasites Wildl. 2020b, 13: 196-201.
7. Jiang Y, Xin S, Ma Y, Zhang H, Yang X, Yang Y. Low Prevalence of *Toxoplasma gondii* in sheep and isolation of a Viable Strain from Edible Mutton from Central China. Pathogens (Basel, Switzerland), (2023).12(6), 827.
8. Li YN, Nie X, Peng QY, Mu XQ, Zhang M, Tian MY, Min SJ. Seroprevalence and genotype of *Toxoplasma gondii* in pigs, dogs and cats from Guizhou province, Southwest China. Parasit Vectors. 2015, 8:214.
9. Liang Y, Chen J, Meng Y, Zou F, Hu J, Esch GW. Occurrence and genetic characterization of GRA6 and SAG2 from *Toxoplasma gondii* oocysts in cat feces, Kunming, China. Southeast Asian J Trop Med Public Health. 2016, 47(6):1134-1142.
10. Qian W, Wang H, Su C, Shan D, Cui X, Yang N, Lv C, Liu Q. Isolation and characterization of *Toxoplasma gondii* strains from stray cats revealed a single genotype in Beijing, China. Vet Parasitol. 2012, 187(3-4): 408-413.
11. Ren H, Yang L, Zhu N, Li J, Su C, Jiang Y, Yang Y. Additional evidence of tigers (*Panthera tigris altaica*) as intermediate hosts for *Toxoplasma gondii* through the isolation of viable strains. Int J Parasitol Parasites Wildl. 2022, 19:330-335.
12. Su R, Dong H, Li T, Jiang Y, Yuan Z, Su C, Zhang L, Yang Y. *Toxoplasma gondii* in four captive kangaroos (*Macropus* spp.) in China: Isolation of a strain of a new genotype from an eastern grey kangaroo (*Macropus giganteus*). Int J Parasitol Parasites Wildl. 2019, 8: 234-239.

13. Wang L, Chen H, Liu D, Huo X, Gao J, Song X, Xu X, Huang K, Liu W, Wang Y, Lu F, Lun ZR, Luo Q, Wang X, Shen J. Genotypes and mouse virulence of *Toxoplasma gondii* isolates from animals and humans in China. PLoS One. 2013a, 8(1): e53483.
14. Wang L, Cheng HW, Huang KQ, Xu YH, Li YN, Du J, Yu L, Luo QL, Wei W, Jiang L, Shen JL. *Toxoplasma gondii* prevalence in food animals and rodents in different regions of China: isolation, genotyping and mouse pathogenicity. Parasit Vectors. 2013b, 6: 273.
15. Wang D, Liu Y, Jiang T, Zhang G, Yuan G, He J, Su C, Yang N. Seroprevalence and genotypes of *Toxoplasma gondii* isolated from pigs intended for human consumption in Liaoning province, northeastern China. Parasit Vectors. 2016, 9: 248.
16. Xin S, Jiang N, Yang L, Zhu N, Huang W, Li J, Zhang L, Su C, Yang Y. Isolation, genotyping and virulence determination of a *Toxoplasma gondii* strain from non-human primate from China. Transbound Emerg Dis. 2022, 69(2): 919-925.
17. Yang L, Xin S, Zhu N, Li J, Su C, Yang Y. Two viable *Toxoplasma gondii* isolates from red-necked wallaby (*Macropus rufogriseus*) and red kangaroo (*M. rufus*). Parasitol Int. 2023a, 92: 102687.
18. Yang L, Ren H, Zhu N, Mao G, Li J, Su C, Jiang Y, Yang Y. Epidemiology and isolation of viable *Toxoplasma gondii* strain from macropods. Heliyon. 2023b, 9(3): e13960.
19. Yang Y, Dong H, Su R, Li T, Jiang N, Su C, Zhang L. Evidence of red panda as an intermediate host of *Toxoplasma gondii* and *Sarcocystis* species. Int J Parasitol Parasites Wildl. 2019a, 8: 188-191.
20. Yang Y, Dong H, Su R, Jiang N, Li T, Su C, Yuan Z, Zhang L. Direct evidence of an extra-intestinal cycle of *Toxoplasma gondii* in tigers (*Panthera tigris*) by isolation of viable strains. Emerg. Microbes Infect. 2019b, 8(1): 1550–1552.
21. Yang Y, Jiang N, Xin S, Zhang L. *Toxoplasma gondii* infection in white spoonbills (*Platalea leucorodia*) from Henan Province, China. Emerg Microbes Infect. 2020, 9(1): 2619-2621.
22. Yang YR, Feng YJ, Lu YY, Dong H, Li TY, Jiang YB, Zhu XQ, Zhang LX. Antibody detection, isolation, genotyping, and virulence of *Toxoplasma gondii* in captive felids from China. Front Microbiol. 2017a, 8: 1414.
23. Yang Y, Feng Y, Yao Q, Wang Y, Lu Y, Liang H, Zhu X, Zhang L. Seroprevalence, isolation, genotyping, and pathogenicity of *Toxoplasma gondii* strains from sheep in China. Front Microbiol. 2017b, 8: 136.
24. Yang Y, Ying Y, Verma SK, Cassinelli AB, Kwok OC, Liang H, Pradhan AK, Zhu XQ, Su C, Dubey JP. Isolation and genetic characterization of viable *Toxoplasma gondii* from tissues and feces of cats from the central region of China. Vet Parasitol. 2015, 211(3-4): 283-288.
25. Zhou Y, Zhang H, Cao J, Gong H, Zhou J. Isolation and genotyping of *Toxoplasma gondii* from domestic rabbits

in China to reveal the prevalence of type III strains. Vet Parasitol. 2013, 193(1-3): 270-276.

26. Zhou P, Zhang H, Lin RQ, Zhang DL, Song HQ, Su C, Zhu XQ. Genetic characterization of *Toxoplasma gondii* isolates from China. Parasitol Int. 2009, 58(2): 193-195.

27. Zhang W., Liu J.S., Ma Y.W., Lu S. 1987. *Toxoplasma gondii* isolated from a dead deformed fetus. Chinese Journal of Zoonoses, 3, 26 (in Chinese).
